# A discussion of multiculturalism in Australia from educators’ perspective

**DOI:** 10.1186/2193-1801-3-36

**Published:** 2014-01-17

**Authors:** Lily A Arasaratnam

**Affiliations:** Alphacrucis College, 30 Cowper Street, Parramatta, NSW2150 Australia

**Keywords:** Multiculturalism, Australian educators, Thematic analysis

## Abstract

This study is an exploration of the views of non-indigenous Australian tertiary educators (N = 22) on multiculturalism and its effects on Australian society. Thematic analysis was used to identify four dominant themes in interviews conducted with the participants. The results are discussed within the themes of Australian identity, attitudes toward multiculturalism, the role of education/educators in multiculturalism, and the future of multicultural Australia. The results reveal that, while there are strong concerns about current policies, tertiary educators have favourable views toward multiculturalism and believe that multicultural views should be integrated into the curriculum from primary education onwards. Despite growing cultural diversity, results further indicate that there is a gap between the ideology and the practice of multiculturalism in Australia.

“We speak over 260 languages and identify with over 270 ancestries. Australia is and will remain a multicultural society” (Australia’s Multicultural Policy, p. 2). Urban Australia is unquestionably multicultural and fluid in its social composition. Despite varying opinions for or against immigration and its effects on the cultural diversity of Australia, the fact remains that cultural plurality is everyday reality in Australian urban cities and the inhabitants of these cities, for the most part, seem to navigate the rapidly changing social landscape with relative ease.

While familiarity with cultural diversity has fostered an environment of amicable co-existence in urban Australia, the influx of immigrants (particularly asylum seekers) and the growth of politically vocal minorities have contributed to social tension, which has been particularly significant since the formative events of 9/11. Ambivalence or even hostility toward certain kinds of immigrants is not unusual in Australian history, as evident in the anti-Asian immigration policies of the former One Nation Party. On the one hand there are those who feel that Australia has a humanitarian obligation to keep its borders open to asylum seekers (Hugo 2002a) and to consider the broader global implications of policies pertaining to asylum seekers (Yeatman 2003) and on the other hand there are those who believe that strong border policies are necessary for building secure communities (Betts 2001). McMaster (2002) observes that Australia’s reputation as a tolerant society and its recent policies on mandatory detention for asylum seekers contribute to a paradox that “reflects a sense of insecurity that seems to be embedded in the Australian psyche” (p. 279).

The attitude toward asylum seekers is just one aspect of how contemporary issues relating to cultural diversity are affecting Australian society and Australian identity. While several studies have addressed multiculturalism in Australia (Forrest and Dunn 2010; Jakubowicz 2011; Liu 2007; Sakurai et al. 2010), it is informative to explore this topic from the perspective of Australians who are providers of tertiary education because these individuals are arguably in a position to influence mindsets of future policy-makers, and presumably have the skills to discuss these topics with reasoned thought. To this end, this study presents the opinions of non-indigenous Australian educators in which the topics of multiculturalism and Australian identity are discussed.

## Cultural diversity in Australia

Australia is a nation made up of Aboriginal Australians and a myriad of immigrants, some of the earliest of whom trace their ancestry to Britain (for a brief history of Australian immigration and policies post WWII, see Poynting and Mason 2007). While Australians’ attitudes toward immigrants have varied over the years, Pietsch and Marotta (2009) note certain patterns in these attitudes. For example, Australians have been generally positive toward the idea of skilled migrants, but prefer European migrants to Asian or Middle Eastern migrants; this preference was further aggravated by the events of 9/11. One outward manifestation of this tension was the 2005 Cronulla riot in New South Wales (Lattas 2007; Redmond 2007; Scott 2006). This event is seared in the psyche of modern-day Australians, as is evident in the reflections of several of the participants in the present study. Further debates of asylum-seekers and “boat people” in present-day Australia are continuing to contribute to ambivalent attitudes toward cultural diversity, at least among some Australians. There is also evidence to show that Australian government policies have become increasingly stricter in regards to immigration (see Hugo 2002b).

Regardless of whether one is for or against cultural diversity in Australia, the fact remains, Australian identity and Australian policies are being shaped by cultural diversity. Australia’s Multiculturalism Policy exemplifies an effort by the government to formalise the ideology that is meant to undergird the everyday social interactions among Australians (Arber 2006). According to an early definition of multiculturalism proposed by Dolce (1973), multiculturalism is “a reflection of a value system which emphasizes acceptance of behavioural differences deriving from differing cultural systems and an active support of the right of such differences to exist” (p. 283). Though there is arguably a gap between ideology and practice, the formalisation of a multicultural policy is a step toward “active support” of cultural differences. Meanwhile however, media coverage of issues relating to asylum seekers and Muslim immigrant groups presents a sense of ambivalence toward that which is different and unknown. The dehumanisation of “boat people” is a subject that pertains to a wider discussion well outside the borders of Australia (Pugh 2004). In the Australian context, media coverage of asylum seekers has created much political and social debate in recent times (Pickering 2001). O’Doherty and Lecouteur (2007) observe that labels such as “boat people” and “illegal immigrants,” that are often used by media, are blurring into a category which is in essence a category of “undesirable” arrivals.

In addition to the social debates about asylum seekers, the Australian Islamic community has also been of interest in recent media coverage. Rane et al. (2011) note that the opinions and values of Australians Muslims are vastly different from the stereotypical portrayal of this group in the media, observing that Muslims in Western societies are working toward harmony and understanding their faith in the context in which they find themselves. While it is not the purpose of this study to focus solely on attitudes toward particular immigrant groups, the matter of asylum seekers and attitudes toward Muslim immigrants are noteworthy because these are topics that have occupied much of Australian social and political conversations in recent years, and are thus pertinent to any discussion of multiculturalism in Australia.

Van de Vijver et al. (2008) posit that multiculturalism can refer to three things; namely a demographic feature, a label to identify policy regarding cultural diversity, or an attitude toward the ideology of many cultures co-existing in mutual acceptance. The aspect of multiculturalism that is most central to the present study is the latter. That is, the present study focuses on a discussion of attitudes and opinions about multiculturalism, based on the participants’ experiences of the demographic and policy-related facets of multiculturalism.

### Rationale for the study

As previously noted, not many studies of multiculturalism in Australia have focused on exploring tertiary educators’ view on the subject. Tertiary education providers, particularly lectures, regularly interact with students who have chosen to further their formal education toward intellectual and career development. This group is also arguably poised to be policy makers and/or opinion leaders. While it is not yet unequivocally clear whether tertiary education fosters inclusive attitudes toward multiculturalism, it is empirically clear that lesser education is associated with xenophobia and an exclusive view of the Australian identity (Phillips 1998). It is interesting to explore the attitudes and opinions held by the lecturers with whom the students in tertiary education interact. Further, lecturers often experience cultural diversity on a regular basis in the classroom, particularly considering the large number of international students who choose to pursue higher education in Australia.

Lecturers in tertiary education also often hold higher education qualifications, by nature of their profession, and are hence trained in systematic and critical thinking that helps them to identify certain social patterns. Zerubavel (1997) calls this *optical socialisation*, the process where professionals are trained to see things that may not be obvious to the untrained eye. Having completed at least an undergraduate degree (and in most cases, one or more postgraduate degrees), lecturers in tertiary education are such a group of individuals who are trained in systematic and critical thinking, and are arguably able to identify social patterns and phenomena that others may not observe. Hence the observations made by this group of people may provide important insight into multiculturalism in Australia.

Participants were chosen on the basis of them being non-indigenous but either born in Australia or migrated to Australia before the age of one. The rationale for the choice was that a non-indigenous Australian is an immigrant (at least by ancestry), and therefore arguably has no special claim to the land compared to more recent immigrants. However, as someone born in Australia (or raised locally as an infant), such a person might have a well-formed identity as an Australian who belongs to the land, and may view recent immigrants from other countries as the “outsiders.” A multicultural Australia, however, is based on the ideology of equal rights and claims for all Australian citizens, regardless of their ancestry or immediacy to immigration. Discovering whether or not this ideology is shared among non-indigenous Australians is part of the purpose of the present study.

## Method

### Participants

Approval for this study was obtained from Alphacrucis College Research Ethics Committee prior to data collection. The participants were non-Aboriginal Australian tertiary educators (N = 22, Male = 16, Female = 6, Average age = 43.86). The participants were currently or previously involved in providing tertiary education, and represented 7 different institutions in Sydney and a variety of fields of expertise, such as music, sociology, history, theology, Biblical studies, leadership, business, education, and nursing. Most participants were from Anglo-Saxon and Anglo-Celtic backgrounds, with the exception of two who were of mixed ancestry (namely European-Asian).

### Procedure

The participants were selected based on personal contact with the researcher and through referrals. Each participant was given the opportunity to provide informed consent. Data collection involved an interview, either face-to-face or via telephone (average time = 42 minutes). Each participant was asked to describe what it means to be Australian, what multiculturalism means to them, and to comment on multiculturalism in Australia, particularly from the perspective of a tertiary education provider. The interviews were audio-recorded (with the participants’ consent) and transcribed.

### Analysis

The method chosen for analysing the transcripts is thematic analysis. This method was chosen because, while allowing for dominant themes to be extracted from the transcripts, thematic analysis provides the researcher to uncover these themes without necessarily being driven by a pre-existing theoretical framework (Braun and Clarke 2006). It is necessary, however, to explicate certain assumptions on which this analysis is based. These are as follows: 1) Tertiary education providers, most of whom in the sample having completed doctoral studies, are trained in reasoned thought that is reflected in their responses, 2) Recurring labels in responses to a particular question by multiple participants are indicative of general views on the topic, and 3) Recurring ideas in responses to a particular question, even if expressed in different ways by different participants, are indicative of general views on the topic, at least among tertiary education providers. It must also be noted that, in thematic analysis, the themes are identified not entirely based on the frequency of occurrence but rather on substantive significance. According to Floersch et al. (2010), substantive significance “refers to the consistency of themes across and within study patterns” (p. 408). Hence the data were analysed with the view of identifying dominant themes based on substantive significance. This process, as Floersch et al. note, is also reliant on the researcher’s familiarity with the topic and related literature. The identification of the themes involved the steps of thoroughly familiarising one’s self with the data, then categorising the data into themes, and finally refining the categories based on further analysis of the data. The method of thematic analysis thus relies on the systematic nature of this process and the assumption that findings can be reasonably replicated by another researcher with similar familiarity with the topic, following a similar process.

### Limitations

The results from this study should be understood in the context of certain limitations. First, the opinions of the participants in this study represent those of a very select group of highly educated and arguably privileged section of society, all of whom identified themselves as Christian or Catholic. Therefore their opinions may not be representative of Australians of different faiths. Second, the participants were selected based on their social proximity to the researcher, and as such arguably represent a limited perspective even within the group of tertiary education providers. Third, because of the limited availability of the participants’ time, the interviews were only conducted once. While it must be noted that the participants were given every opportunity to expand on their views and to express their opinions for any length of time during the interview, it is possible that longer and repeated interviews may have extracted details that were not communicated in this study.

## Results and discussion

Four themes were identified in the participants’ responses. These themes are described and discussed in this section.

### Theme I: Australian identity

When asked, “What does it mean to be Australian?” the responses were varied, but certain dominant ideas emerged consistently. These are, freedom, democracy, “fair go” (equal opportunity for all Australians), “mateship” (the notion of lending a hand to one’s neighbour), a love of sports, a love for the outdoors, relaxation and recreation (“beach”), tolerance, authenticity, hard work ethic, diversity, egalitarianism, and an intolerance of pretentiousness (“tall poppy syndrome”). Further, the general consensus among participants was an Australian is someone who identifies with the ethos and culture of Australia, considers Australia to be “home,” regardless of whether s/he lives in Australia or was born in Australia. Some participants noted that, while tolerance is an ideal value in Australia, racism also co-exists. One participant phrased this as, “I think empirically there is just a lot of racism around, but normatively we aspire not to be.”

Common first responses to the question of what it means to be Australian were, “That’s a difficult question!” or “I don’t think anyone has ever asked me that question!” This is possibly indicative of Australia’s infancy as a nation, compared to other older civilisations, and the complexity of the vast array of cultural influences that make up a non-Aboriginal Australian. While the participants were able to identify a variety of labels that they would associate with being “Australian,” the ambiguity associated with what Australian “culture” is, was clear in their wrestling with this question. Based on the participants’ observations (for example, “The European colonisation of Australia imprinted English Caucasian values that were in many ways built on Christian laws and morals”) it was evident that there is indeed a mainstream Australian culture that is heavily influenced by Anglo-Saxon values; however, the participants were unclear as to whether immigration patterns will significantly change this mainstream culture in the decades to come. The words that the participants used to associate with being “Australian” were consistent with their positive views toward multiculturalism (such as “fair go” and “mateship”), and indeed consistent with the dominant paradigm of Australian identity based on literature, commercials, and everyday rhetoric. However, as the participants themselves observed, there is a gap between ideology and practice. In the words of one of the participants of mixed ethnic makeup, referring to a conversation with one of her white co-workers:

He would say oh, you’re not a true Australian, but you’re Australian. But you’re not a true Australian because you don’t look like one. He wasn’t racist in that sense because we’re friends and he’s OK with me. But when it came to defining the term he thinks of Australian as white. I think that’s one thing that’s tricky because people who would call themselves Australian have different ideas of what an Australian is. Nobody really knows and it’s not consistent, there’s no consensus.

The tension between ideology and practice is not unique to Australia. There are studies from multicultural societies in other parts of the world that demonstrate that majority group members’ acceptance of immigrants or minority groups is influenced by the extent to which the latter groups are perceived to be adapting to the “mainstream” culture (Matera et al. 2012; Schalk-Soekar et al. 2004; Tip et al. 2012). The tension between adaptation verses assimilation is reflected in other research as well (Arasaratnam 2013). In a study involving the exploration of “UnAustralian,” Smith and Phillips (2001) note the following:

Our data suggest that ethnic groups…are most likely to be stigmatised as the ‘UnAustralian.’ What is evident is that despite a quarter of a century of official government policy, Australians are still operating within an assimilistic logic…Put simply, the government is not doing a good job of explaining what multiculturalism means in contemporary Australia. (p. 337)

While the participants in the present study were generally favourable toward immigrants and observed that cultural diversity has enriched Australia, it must be noted that these participants represented a select group of highly educated individuals who have had the opportunity to not only interact with people of other cultures on a regular basis but also travel widely. As previously noted, this is not representative of the wider population. It was, however, interesting to explore how the participants’ person attitudes/beliefs about Australian identity and multiculturalism influenced their everyday behaviour. One participant said:

Whenever anybody seems to associate Australian with white Australians I’ll always correct them and go, Australian is a very fluid concept these days. One of the other things that I don’t like is when often ethnic people would say that my nationality is Aussie. As if I intrinsicly have that label. I don’t like that because that just serves to reaffirm the misconceptions.

Other participants similarly expressed that they speak up if they hear racist remarks or that they are intentional seeking out friendships with different types of Australians for the sake of expanding their own understanding. Referring to attitudes of ignorance, one participant said, “All you can do is educate, unfortunately. There’s a limit to education. Knowledge itself doesn’t necessarily change behaviour, perceptions, or strong held beliefs. But I’m in education, I believe in education.”

### Theme II: attitudes toward multiculturalism

The participants unanimously said that they would consider urban Australia to be multicultural. When asked, “What does multiculturalism mean to you?” a common response was, “People of different ethnic backgrounds living together in a geographic location, hopefully in harmony.” One participant described multiculturalism as, “unity without uniformity.” This is again consistent with the participants’ description of Australian identity as egalitarian and, at least in ideology, inclusive of differences.

While the sense of multiple cultures co-existing in a shared space was commonly associated with multiculturalism, the participants also expressed mixed feelings about this concept. The reasons for such mixed feelings ranged from a disappointment in the gap between the ideology of multiculturalism and practical reality, feelings of expectations to compromise one’s values, and perceptions of “clamouring for rights.” One participant observed, “I think multicultural says that there’s a plurality of identities and voices; and I don’t subscribe to the view that this is somehow undermining Australia’s nationhood.” Others however, expressed concern that, in an effort to accommodate multiple voices, the integrity of what it means to be “Australian” could be compromised. This was said specifically in relation to the recent media debates regarding the incorporation of Sharia law into the Australian legal system. One participant noted that a distinction should be made between tolerance and compromise, suggesting that tolerance is what is needed in a multicultural society, but he feels that the minorities are demanding compromise instead.

When asked, “How do you think ‘Australians’ are coping with the influx of immigrants?” the participants’ responses were varied. Out of the 22 participants, two did not quite answer the question, three said that they believe that Australians overall are at ease with cultural diversity and are taking the inclusion of new immigrants into society in stride. One of them observed, “It’s been a bit of a success story, really.”

Five participants responded that they feel Australians are handling the influx of immigrants badly. One of them observed:

…we are multicultural, but we really don’t want to be because what we really want is mono cultural and assimilation rather than integration. They use the word integration to mean assimilation. And certainly that’s what happened in language referring to indigenous culture as well.

The majority (n = 12) had mixed responses to this question. Most of the participants who fell in this group said that the way in which an “Australian” responds to immigrants depends on the extent to which s/he has been exposed to cultural diversity. They observed that most people in urban areas, being used to living in diverse communities, are fairly adept at interacting with immigrants just as they would with anybody else. One participant noted that it is the rate at which communities are becoming diverse (not diversity itself) that is causing unease among Australians. Another participant observed, “In a sense this is a funny question because the statistics are over 50% of the population is either born overseas or their parents were born overseas. So who’re the Australians who are coping?”

Most participants expressed that the advantages of a multicultural society outweigh the disadvantages. Of the advantages, the participants noted that diversity brings a wider understanding of the world to Australians, an opportunity to interact with people who are different, and the access to a variety of cultural and culinary experiences. Further, some participants observed that close interactions with people from a variety of cultural backgrounds gives Australians a competitive advantage in global business and economic ventures.

Of the disadvantages, the participants highlighted conflicts of values. For example, several participants noted that the values of Muslim immigrants are often in conflict with what they would view as Australian values, such as equal rights for women. Interestingly, several participants, when asked why there seems to be particular tension with the Muslim community in comparison to the myriad of other religious groups that seem to have harmoniously blended into Australian society, expressed that there is a perception of Muslims as being fundamentalists, and as such, they grate against values of Australians who inherently resist fundamentalism.

Another observation was that immigrant groups that have previously existing conflicts should not bring those conflicts to Australia, if they choose to migrate to Australia. In one participant’s words:

I don’t think it works when people come to Australia and decide to be *in* Australia but not at all *of* Australia. It’s like Australia doesn’t exist, but they want to be here. So they bring their political issues, their seclusions, and there’s not really a willingness to embrace more than just the land. I don’t think that works because if anyone comes to Australia, as an Australian I actually want to engage with them.

While all participants expressed that immigrants should maintain their own cultural values to a certain extent, they were also unanimous in their view that a multicultural society is not one that is made up of ghettos of migrants but rather one in which there is regular interaction between different groups of people and that whoever chooses to live in Australia should abide by the laws of the land. They further asserted that because English is the dominant language in Australia, it is necessary for immigrants to learn English in order for them to fully participate in Australian society.

### Theme III: the role of education/educators in multiculturalism

While participants were unanimous in their opinion that higher education has a role to play in influencing and educating people about cultural diversity, many noted that exposure to diversity should begin in primary education and informal conversations at home and that multicultural education should be incorporated into the system, but not necessarily as a stand-alone subject. One participant observed that if multicultural education is a stand-alone subject, then this draws attention to it. The values of multiculturalism should instead be organically integrated into the whole curriculum.

In regards to higher education, the participants expressed that one of the goals of higher education is to train students in critical thinking skills. As such, higher education equips students to engage with multiple points of view and critically examine social messages without passive consumption. Some of the participants also noted that, because of the large number of international students who come to Australia for tertiary education, Australian students are often first exposed to cultural diversity in a real way in a university or college classroom. Several of the participants expressed that this was indeed their own experience.

Because the participants were education providers, it was interesting to explore their extent to which they communicate their views on multiculturalism (implicitly or explicitly) to their students. One of them said, “sometimes I would see students generalising other cultures and so I try to be careful to make sure that we make a distinction that we’re not referring to everybody in that we can talk about distinctions and generalisations;” another participant explained that, when teaching, she made a conscious effort to use illustrations that are accessible to a variety of students instead of relying on ‘typical’ Australian cultural references with which she was familiar; several of the participants expressed awareness of varying language abilities of their students and that they make allowances for students whose first language is not English by annunciating clearly and repeating things that might be harder to understand. These observations illustrate the ways in which the participants’ beliefs about multiculturalism influence their behaviour in the classroom.

Apart from observing that tertiary education is critical in exposing people to different worldviews and equipping them with critical thinking skills, this group of educators did not have much to say on the subject of *how* positive attitudes toward multiculturalism can be fostered in the tertiary educational setting. They generally agreed that, while education can provide the right thinking tools, positive exposure to cultural diversity is more effective in fostering inclusive attitudes. Further, other research suggests that such exposure might increase one’s cognitive complexity (Benet-Martinez et al. 2006) and cognitive complexity, in turn, is associated with the ability to deconstruct stereotypes in positive ways (Arasaratnam 2011). The provision of fee-help and other such financial means for a larger number of the population to access higher education is positive, given the opportunities this provides for exposure to different ideas and people from different cultures. However, Swetnam (2003) notes that further steps can be taken in education, such as multiculturalism training for teachers, hiring culturally diverse teaching staff, and integrating multicultural content into the curriculum.

### Theme IV: the future of multicultural Australia

When asked, “What would you like to see in Australia in ten years’ time?” there were three types of responses. Firstly, the participants expressed a hope for acceptance and integration of people of different cultures; secondly, a positive change in the general populations’ attitude toward immigration and immigrants, reflected in changes in policy as well; and thirdly, the resolution of the current issues of antagonism toward Muslims and asylum-seekers.

The participants observed that in a decade’s time, the opinion-leaders may be first or second-generation children of migrants who are comfortable with diversity and would not make a big issue of what seem like issues in the current social climate. One participant noted that the integration of diversity should be reflected in parliament representation: “If you look at parliament house, they are all white men predominantly. So where are the other voices and cultures?” The participants also expressed that they would like to see much more “mixing of cultures” and less of ghettos.

The participants noted that Australia needs immigration, and that migration of people from war-torn or other nations to Australia is not going to diminish in the coming years. Hence, regardless of the origin of the immigrants, there needs to be an understanding of the country’s dependence on immigration and the recognition that immigration is part of ongoing reality in Australia. One participant observed that the patterns of attitude toward immigrants are repeating themselves, in that the attitudes that were once directed toward Italian and Greek immigrants are now directed toward Muslim and certain Asian immigrants. There was general discontent about current immigration policies.

The issues relating to asylum seekers and Muslims were at the forefront of the participants’ responses to this question, reflecting the current media coverage of these issues. The participants expressed hope that in a decade’s time these would not be issues and that Australians would have found a way to move beyond what they perceived as petty or ignorant attitudes. One participant said, “Secretly I’m hoping for a bit of civil disobedience on those sort of issues; because the rhetoric is getting harsher and it could lead, policywise, to some scary situations.”

The general hope that the participants expressed for a more inclusive Australia in ten years’ time was tempered with concern that patterns in history seem to be repeating themselves, and therefore they may still do so in a decade’s time. One of the sentiments commonly expressed was that the participants felt “lucky” or “blessed” to be Australian. While communicating their views on what they hoped to see in Australia in the next decade, they prefaced their responses by noting that their opinions were based on their own experiences, most of which were from the point of view of what would be considered privileged, middle-class, “mainstream” Australian.

If Australia is committed to multiculturalism, then, as Smith and Phillips (2001) observe, the Australian government needs to take more deliberate steps toward bridging the gap between ideology and practice. This could start in the education sector, but community initiatives are necessary as well. The participants in this study suggested some steps towards this, such as providing opportunities for cultural and language exchange in community programs, and wider communication of multicultural ideology in implicit ways such as diverse representations in government bodies, media, and education. It must be noted, however, that community initiatives such as Global Backyard that was previously mentioned and the Welcome to Australia program that is dedicated to fostering communities that are open to cultural diversity (http://www.welcometoaustralia.org.au/), illustrate that interest in a multicultural Australia may not be entirely limited to political rhetoric or the educational sector.

## Conclusion

The purpose of this study was to explore opinions on multiculturalism in Australia, of non-indigenous Australian tertiary education providers. This group was selected based on the rationale that, being highly educated individuals, this group of people may be able to provide reasoned articulation of opinions on multiculturalism from the perspective of Australians who are themselves descendents of immigrants. Thematic analysis of interview data highlighted four themes. It was generally evident that the participants recognised Australia to be a multicultural country and that this is a positive thing. Additionally, the results show that there is desire among tertiary education providers to integrate multicultural views into the education system throughout, starting with primary education. There is also a concern for the way in which certain immigrant groups have been marginalised in media coverage, and a concern about the current inadequacy of immigrant policies to deal with asylum seekers in a fair and humane way.

If Australia is committed to multiculturalism, then there is a pressing need for deliberate measures to foster attitudes of inclusion and cohesion, particularly including multiple cultural representations in the construction of Australian identity. As one of the participants observed, places of worship provide infrastructure in which diverse people who share the same faith could gather. This is an example of a social instance in which multiculturalism is facilitated by shared values and goals. There are also other initiatives that are already facilitating integration of immigrants into the Australian society and educating Australians on multiculturalism. For example, the non-profit organisation Global Backyard connects new immigrants (particularly refugees) with Australian volunteers in mentoring programs and facilitates skills development and procurement of employment (Mamamia Team 2011). The provision of such opportunities in the wider community through programs and organisations that emphasise common goals is necessary, as perceived similarity attracts people to one another, even amidst cultural diversity (Osbeck et al. 1997).

If we are to get a glimpse of the attitudes that are being communicated to students in tertiary classrooms based on the results of this study, it can be concluded that the students are being challenged to be inclusive of cultural diversity, open to engaging with people who are different, and to critically examine ideas put forth by the media. Perhaps these are not unusual or even new findings. However, the results from this study present an alternative perspective to what could be perceived as the dominant paradigm. To elaborate, media coverage of asylum seekers and tensions between Islamic values and the broader Australian values arguably generate a perception that the majority of Australians are anxious about new immigrants, concerned about the infringement of other cultures on what is “Australian,” and are generally negatively pre-disposed toward cultural diversification of Australia. However, there are highly educated Australians who feel that government policies should facilitate the integration of asylum seekers into society, and who are able to identify an Australia in which core values do not preclude the harmonious co-existence of multiple cultures. If this group of individuals is able to communicate their values to the next generation of Australian policy-makers and opinion leaders, then perhaps, in the years to come, Australia will be closer to bridging the gap between the ideology and the practice of multiculturalism.
